# The Effects of Early Life Stress on the Brain and Behaviour: Insights From Zebrafish Models

**DOI:** 10.3389/fcell.2021.657591

**Published:** 2021-07-21

**Authors:** Helen Eachus, Min-Kyeung Choi, Soojin Ryu

**Affiliations:** ^1^Living Systems Institute and College of Medicine and Health, University of Exeter, Exeter, United Kingdom; ^2^Institute of Human Genetics, University Medical Center, Johannes Gutenberg University Mainz, Mainz, Germany

**Keywords:** zebrafish, early life stress (ELS), HPA axis, behaviour, brain development

## Abstract

The early life period represents a window of increased vulnerability to stress, during which exposure can lead to long-lasting effects on brain structure and function. This stress-induced developmental programming may contribute to the behavioural changes observed in mental illness. In recent decades, rodent studies have significantly advanced our understanding of how early life stress (ELS) affects brain development and behaviour. These studies reveal that ELS has long-term consequences on the brain such as impairment of adult hippocampal neurogenesis, altering learning and memory. Despite such advances, several key questions remain inadequately answered, including a comprehensive overview of brain regions and molecular pathways that are altered by ELS and how ELS-induced molecular changes ultimately lead to behavioural changes in adulthood. The zebrafish represents a novel ELS model, with the potential to contribute to answering some of these questions. The zebrafish offers some important advantages such as the ability to non-invasively modulate stress hormone levels in a whole animal and to visualise whole brain activity in freely behaving animals. This review discusses the current status of the zebrafish ELS field and its potential as a new ELS model.

## Introduction

Stress disrupts homeostasis and drives an adaptive response, which protects an organism from damage (Chrousos, 2009). However, excess stress or stress during a vulnerable period can impose high allostatic load, which ultimately drives or accelerates maladaptive processes (De Kloet et al., 2005; Danese and McEwen, 2012; Chen and Baram, 2016). In humans, experience of early life stress in the form of childhood abuse or neglect (collectively called adverse childhood experiences or ACE) is associated with the development of behavioural abnormalities and psychiatric disorders in adulthood (Heim and Nemeroff, 2001; Bale et al., 2010; Kessler et al., 2010; Nemeroff, 2016). Further, for mood and anxiety disorders such as depression there is evidence that the course of illness is more severe in patients who experienced ELS, and that these patients are also less responsive to treatment (Nanni et al., 2012).

Whilst the effects of stress in adulthood can be transient and reversible, stress during early life is associated with an alteration of the developmental trajectory of the brain, which can lead to long lasting behavioural alterations (Danese and McEwen, 2012; Bick and Nelson, 2016; Teicher et al., 2016; Agorastos et al., 2019). Behavioural symptoms resulting from ELS exposure may develop during childhood or adolescence or they can be adult-onset. The latter might occur when stress modifies the developmental trajectory of the brain leading to an ‘incubation period,’ such that the effects may not be apparent acutely but may only emerge later once development is complete (Lupien et al., 2009). In support of this, the average time from abuse onset to development of depression in humans is more than 10 years, with symptoms often emerging during adolescence (Spatz Widom et al., 2007).

This remarkable plasticity of the developing brain also means that early intervention has huge potential to prevent the development of adverse phenotypes caused by ELS (Veenstra-VanderWeele and Warren, 2015; Bick and Nelson, 2016). However, we currently lack a thorough mechanistic understanding of how molecular consequences of ELS translate into adverse phenotypes in adulthood. In order to investigate the underlying mechanisms of ELS, various rodent models have been used widely in the past decades. This vast literature has addressed the neuroendocrine and immunological consequences of ELS (Agorastos et al., 2019); structural and functional changes to the brain (Fumagalli et al., 2007; McEwen et al., 2016); the role of gene-environment interactions in modulating disease vulnerability (Klengel and Binder, 2015); and the role of epigenetic mechanisms in mediating the effects of ELS on disease risk (Maccari et al., 2014), including their role in transgenerational stress inheritance (Bale, 2015), amongst others. Yet some questions have been difficult to address in rodent models such as whole brain anatomical and functional alterations induced by ELS. In recent years, a non-mammalian vertebrate model system, zebrafish, was introduced as a new model for ELS studies.

Zebrafish were first used as a vertebrate developmental model for studying gene functions and have been very successful in advancing these fields (Grunwald and Eisen, 2002; Holtzman et al., 2016). The attributes of the zebrafish that lend themselves well to developmental studies, such as the transparent larva, external and rapid development of the embryo, significant homology with other vertebrates, and genetic tractability, also make zebrafish well suited as an ELS model. On top of this, zebrafish offer some practical advantages over mammalian models, such as their small size, ease of breeding in large numbers and lower maintenance cost. In more recent years, advancements such as the ease of genetic manipulation via CRISPR (Li et al., 2016; Albadri et al., 2017), optogenetics (Arrenberg and Driever, 2013) and live whole-brain imaging in freely behaving animals (Portugues et al., 2014; Orger and De Polavieja, 2017) make the zebrafish a particularly attractive model with which to study molecular mediators of behaviour. Lastly, as a vertebrate, zebrafish possess a stress response system with high degree of conservation to the mammalian system. In particular, zebrafish possess the Hypothalamo-Pituitary-Interrenal (HPI) axis, which is the homologue of the Hypothalamo-Pituitary-Adrenal (HPA) axis of the mammalian system. Together, these features make zebrafish highly suitable as an ELS model, and in particular offer potential for detailed analysis of the underlying molecular mechanisms by which ELS affects the brain and behaviour and for drug discovery.

The zebrafish also exhibits some important differences with mammals, which should be carefully considered when selecting an animal model for ELS studies. The zebrafish embryo develops externally, and receives no significant parental care. As such, the effects of prenatal stress during gestation cannot be studied, and the maternal influence to the offspring is limited. However, the accessibility of the early embryo makes the zebrafish a suitable choice for early developmental studies and exposure to chemical compounds during early life can be achieved in a controlled and convenient manner. Unlike in rodents, naturally occurring variation in maternal care cannot confound the early life experience of zebrafish larvae, allowing for a more uniform rearing environment among individuals. Another consideration is that teleost fish have undergone a genome duplication event, and as such zebrafish possess two copies of some genes that are present in only a single copy in mammals (Howe et al., 2013). In some cases this could make it more difficult to understand a gene’s function in zebrafish. However, in some cases, such as for the genes coding for the HPA axis components corticotrophin-releasing hormone (Crh), adrenocorticotropic hormone (ACTH), and glucocorticoid receptor (GR), only a single functional gene exists in zebrafish (Alsop and Vijayan M., 2009). Meanwhile in other cases, two copies of a gene may be advantageous to study the function of a gene for which a null mutation in mouse is lethal.

The aim of this review is to discuss the current status of the zebrafish ELS field and its potential as a novel ELS model. Here, we discuss the recent results obtained from various zebrafish ELS models including prenatal and maternal stress models, models of foetal alcohol syndrome, and effects of early life stressor exposure. We discuss at length the lessons gained from HPI axis manipulation using genetic, optogenetic or pharmacological manipulation in zebrafish. We begin each subsection of this review with a discussion of lessons learned from rodent models, in terms of what is known about the effects of ELS on the brain and behaviour and the molecular players that are implicated and then discuss findings from the zebrafish work. The goal is to provide relevant rodent studies as a background for zebrafish studies rather than providing an exhaustive review of rodent ELS studies, which is outside the scope of this work. For this the readers are referred to several excellent recent reviews on this topic (Maccari et al., 2014; Bale, 2015; Suri and Vaidya, 2015; Di Segni et al., 2018). This review attempts to summarise and provide a contextual framework for the contributions made by the zebrafish work so far and highlights major issues for future work in this exciting and burgeoning field.

## The Stress Response in Zebrafish

The zebrafish is known to respond to a variety of stressors such as chemical (Egan et al., 2009) and physical cues, manipulations of the rearing/housing environment. Measuring the endocrine response to stress in terms of cortisol level is possible in both adult and larval zebrafish (Cachat et al., 2010; Yeh et al., 2013) and larval zebrafish can respond to an acute stressor via an elevation of cortisol level as early as 3–4 days post fertilisation (dpf) (Alsop and Vijayan, 2008; Alderman and Bernier, 2009). Other tools that can be used as stress sensors are also available in fish, such as transgenic lines that can detect stressors that alter motor function (Shahid et al., 2016) or alter glucocorticoid (GC) signalling (Weger et al., 2013; Krug et al., 2014), and behavioural assays to identify HPI axis modifiers in the rapid time domain (Lee et al., 2019). Larval zebrafish are known to alter their behaviour in response to an acute stressor (De Marco et al., 2013, 2016; Lopez-Luna et al., 2017; Lee et al., 2019) and following exposure to a physical stimulus (Castillo-Ramírez et al., 2019). Zebrafish larvae display a variety of behaviours that can be quantified (Kalueff et al., 2013; De Marco et al., 2014, 2016; Groneberg et al., 2015; Karpenko et al., 2020) and exhibit social behaviour (Engeszer et al., 2007; Dreosti et al., 2015; Groneberg et al., 2020), some of which can be modulated by stressor exposure (Eachus et al., 2017; Shen et al., 2020). In the adult zebrafish, a wide variety of behavioural paradigms have been established including tests for anxiety (Egan et al., 2009; Maximino et al., 2010; Stewart et al., 2012), social behaviour (Green et al., 2012; Carreño Gutiérrez et al., 2019), fear (Faustino et al., 2017), and aggression (Oliveira et al., 2011; Carreño Gutiérrez et al., 2018). Adult zebrafish also modulate their behaviour in response to stress, and a variety of stress-sensitive behavioural assays are established (Egan et al., 2009; Clark et al., 2011; Kalueff et al., 2013).

In addition to the genome and brain patterning, zebrafish share extensive homologies with humans in terms of the stress regulation system (Alsop and Vijayan M., 2009). The anatomical centres and molecular components that make up the fish HPI axis are highly conserved among vertebrates, with the interrenal gland representing the fish counterpart of the mammalian adrenal gland (Alsop and Vijayan M.M, 2009). In fish, stress signals are processed in the nucleus preopticus (NPO), the analogue of the mammalian paraventricular nucleus (PVN), of the hypothalamus, which exhibits a remarkable similarity in general anatomy and organisation with other vertebrates (Herget et al., 2014; Nagpal et al., 2019). Parvocellular neurons of the NPO directly innervate the rostral pars distalis of the pituitary gland (Nagpal et al., 2019), where, upon stress exposure, the neurohormone Crh is released. Crh binds its receptor in corticotroph cells of the anterior pituitary, which results in the production and secretion of ACTH (adrenocorticotropic hormone), which reaches the interrenal gland via the circulatory system (Alderman and Bernier, 2009). Here ACTH binds its receptor, melanocortin 2 receptor (MC2R). As in humans, steroidogenic cells of the interrenal gland then synthesise and secrete the main stress hormone cortisol. Cortisol acts via the mineralocorticoid receptor (MR) and glucocorticoid receptor (GR), where ligand binding alters transcription of target genes and exerts negative feedback to the HPA axis at the level of the hypothalamus and pituitary (Charmandari et al., 2004). An important feature of the stress response system in mammals is the existence of a Stress Hyporesponsive Period (SHRP) during early life, when the HPA axis can only be activated by a life-threatening stimulus. This is thought to function to protect the body’s systems, particularly the developing brain, from the deleterious effects of cortisol. It has been suggested that the first 3–4 days of embryogenesis in zebrafish may represent the SHRP (Steenbergen et al., 2011), yet it remains to be determined whether this represents a true SHRP, as the larval HPI axis is still developing at that stage.

## Prenatal Stress

### Prenatal Stress Models in Rodents

During the earliest stages of development when the mammalian embryo or foetus develops *in utero*, the foetus can be indirectly subjected to changes in the external and internal environment of the mother via the placental blood supply. Stressor exposure during pregnancy therefore has the potential to be transmitted to the developing offspring, particularly via maternal-foetal cortisol transfer. It is suggested that the prenatal period may be the most stress-sensitive developmental period, since during this time the foetus is undergoing rapid development. Studies in humans suggest that stress exposure during different gestational time points is associated with alterations in the specific structures or systems that are developing during that period (Lupien et al., 2009; Heim and Binder, 2012; Bick and Nelson, 2016; Teicher et al., 2016). In humans, stress during pregnancy is associated with increased risk of neurodevelopmental disorders and mental health problems in the offspring (Beydoun and Saftlas, 2008; Cottrell and Seckl, 2009; Babenko et al., 2015). Animal models suggest that the HPA axis plays a prominent role in mediating such effects and maternal-foetal cortisol levels are known to show a high degree of correlation (De Kloet et al., 2005; Fumagalli et al., 2007; Cottrell and Seckl, 2009; Nesan and Vijayan, 2013).

One of the most commonly used prenatal ELS paradigms in rodents is prenatal restraint stress (PRS), where the pregnant female is repeatedly restrained during the final week of gestation. This paradigm is known to have long-lasting effects on the brain and behaviour of offspring of PRS dams when tested during adulthood (Maccari et al., 2003, 2014). As adults, offspring of PRS mice have been shown to exhibit hyperactivity, as well as defects in social behaviour, pre-pulse inhibition and fear conditioning (Matrisciano et al., 2013). These behavioural abnormalities could be normalised via administration of valproic acid or clozapine. The offspring of PRS rats have also been shown to exhibit deficits in hippocampus–dependent learning, such as an increased latency to find the platform in a water maze navigation task (Lemaire et al., 2000).

Prenatal restraint stress is known to reduce proliferation of cells in the hippocampus of adult offspring (Lemaire et al., 2006; Belnoue et al., 2013), whilst the effects on neuronal and glial differentiation or neuronal survival are less clear. This effect seems to be region-specific: whilst proliferation is reduced in the dentate gyrus (DG), the olfactory bulb is unaffected (Belnoue et al., 2013). Other studies have suggested that the brains of PRS mice may display epigenetic characteristics similar to those observed in psychiatric patients, such as overexpression of DNA methyltransferase (DNMT) in GABAergic neurons and an increase in 5-methylcytosine and 5-hydroxymethylcytosine in certain CpG-rich regions of the reelin and GAD67 promotors, whose expression was downregulated (Matrisciano et al., 2013).

### Prenatal Stress in Zebrafish

Given the external development of the zebrafish embryo, this animal model offers some unique advantages to study the effects of maternal stress on embryo development and function in later life. Like in mammals, zebrafish females transmit cortisol to the developing embryo, and this is the sole source of cortisol for embryos, until endogenous biosynthesis begins upon hatching (Alsop and Vijayan, 2008). Thus, stressed females may transfer excess cortisol to the developing embryo, which is predicted to affect development. Whilst the study of the effects of maternal stress on offspring in zebrafish is only in its infancy, the results available so far suggest it is a promising model with which to study the mechanisms underlying so-called ‘foetal programming.’

#### Maternal and Paternal Stress

Among the first observations in humans were that prenatal famine exposure and lower birth weight are both associated with cardiovascular and metabolic diseases, as well as cognitive alterations and mental health disorders (Talge et al., 2007; Van den Bergh et al., 2017). This phenomenon can be modelled in zebrafish by exposing adult females to a starvation stress prior to breeding. A relatively short duration of starvation may not affect body size or gross morphology, but can nonetheless have profound effects on the developing brain and behaviour, including hypoactivity (Fan et al., 2019), and suppressed cell proliferation in the forebrain of the early larva (Higuchi, 2020). The mechanisms via which maternal starvation influences offspring development and function are unclear, but given that maternal cortisol levels are increased in zebrafish embryos from starved mothers (Higuchi, 2020) and that cortisol exposure can alter early forebrain neurogenesis in zebrafish larvae (Best et al., 2017) in a similar manner to that seen in the early offspring of starved females, it seems likely that cortisol is a mediator.

Behavioural studies following maternal cortisol exposure in zebrafish have reported a variety of phenotypes including increased boldness (Best et al., 2017) and altered anxiety-like behaviour (Van Den Bos et al., 2019) of offspring. The study by Best et al. (2017) shows that zebrafish larvae exposed to increased maternal cortisol display hyperactivity during the light period of a light-dark assay, and reduced thigmotaxis, or wall-hugging behaviour, in an open field assay. These behaviours, suggestive of increased exploration and reduced anxiety respectively, together indicate a tendency towards increased boldness. Further work has suggested that strain can influence the effects of maternal stress on behaviour in the offspring (Van Den Bos et al., 2019).

The mechanisms underlying behavioural alterations in zebrafish larvae following exposure to increased maternal cortisol are only beginning to be investigated. Initial studies have uncovered alterations in the developing brain, which correlate with the behavioural abnormalities. Best et al. (2017) detected an increase in primary neurogenesis in the preoptic region and pallium, as well as increased brain expression of proneural gene *neurod4* in zebrafish embryos following exposure to maternal cortisol, suggesting that maternal cortisol may alter brain development in a region-specific manner. Additionally, D’Agostino et al. (2019) reported increased basal expression of *cfos* in prenatally stressed zebrafish larvae, as well as altered reactivity of *cfos* expression level to an acute stressor. The *cfos* gene is often used as a marker of neuronal activity, and as such, these data suggest that prenatal exposure to cortisol might affect neuronal function in the basal state, as well as during stimulus processing. Similarly in humans, the offspring of mothers who experienced high levels of anxiety during pregnancy exhibited altered patterns of brain activation during cognitive control tasks (Mennes et al., 2019).

The zebrafish studies described here suggest that the zebrafish system can be used to model the effects of maternal stress and that many of the effects of maternal stress in humans and other animal models can be recapitulated in zebrafish. The zebrafish system offers some advantages over other animal models, including the ability to mimic increased deposition of maternal cortisol under highly controlled conditions, as well as ease of studying the concurrent effects of stress on the developing brain and behaviour in a high-throughput manner. In addition to maternal stress, studies in rodents have demonstrated that paternal stress can also influence behaviour and physiology in the offspring (Yeshurun and Hannan, 2019). This sperm-driven inheritance is thought to be mediated at least in part, by non-coding RNAs (Rodgers et al., 2015). Whilst this area has not yet been well studied in zebrafish, a recent study demonstrated that male zebrafish that were exposed to stress during adulthood had altered levels of several small non-coding RNAs in the spermatozoa, and that their offspring subsequently displayed weakened endocrine and behavioural responses to acute stress (Ord et al., 2020).

#### Models of Foetal Alcohol Syndrome

In addition to transmitting excess cortisol to the developing foetus, human mothers can also expose the foetus to alcohol via the placental blood supply. Animal models, more recently including zebrafish, have been used to model Foetal Alcohol Spectrum Disorder (FASD). FASD is caused by prenatal alcohol consumption, which affects the developing foetus, and has a prevalence of 2–5% in the human population (May et al., 2009). In humans, severe exposure is often associated with gross skeletal and craniofacial abnormalities, and severe central nervous system (CNS) dysfunction, whilst moderate exposure is associated with more subtle cognitive and behavioural defects including depression, vulnerability to stress, impulsivity and inattention and antisocial behaviour. These more moderate cases of FASD are estimated to make up 98% of all cases, and are often left undiagnosed or misdiagnosed (Clarren et al., 2015).

Given the utility of the zebrafish as a shoaling animal, attention has focused on the effects of lower doses of ethanol given during early life on social behaviour. Despite variation in exposure level, developmental timing of exposure, timing and experimental set-up of behavioural assays, several zebrafish studies have reported that ethanol exposure during early development results in impaired social behaviour in adulthood (Fernandes and Gerlai, 2009; Buske and Gerlai, 2011; Parker et al., 2014; Fernandes et al., 2015, 2019a, b). The observed defects in social behaviour were apparent in various contexts, including when an individual fish was presented with the visual cue of a live shoal (Parker et al., 2014), or a computer image (Fernandes et al., 2015), and when fish were observed in a group of conspecifics (Parker et al., 2014). The impairments in social behaviour in zebrafish models of FASD fit well with the reduction of social skills seen in children with FASD (Stevens et al., 2015) and social impairments in mammalian models (Varlinskaya and Mooney, 2014). This suggests that zebrafish models of FASD may be able to provide crucial mechanistic insights into the aetiology of abnormal social behaviour resulting from developmental ethanol exposure.

Although studies have only just begun to investigate the mechanisms underlying impaired social behaviour resulting from developmental ethanol exposure in zebrafish, the results suggest that the dopaminergic neurotransmitter system plays a role. A number of studies have detected a reduction in levels of dopamine and its metabolite, 3,4-Dihydroxyphenylacetic acid (DOPAC), in the brains of adult fish exposed to ethanol during early development, compared with controls (Buske and Gerlai, 2011; Mahabir et al., 2014; Fernandes et al., 2015). Data from Fernandes et al. (2015) suggests that this represents not a reduction in baseline levels, but rather a reduction in the dopaminergic response to a stimulus. If specific to the shoaling stimulus, this could suggest that ethanol-exposed fish find the conspecific cue less rewarding, perhaps due to impaired dopaminergic signalling, leaving exposed fish with a reduced motivation to socialise.

In addition to the defects in neurotransmitter systems following developmental exposure to ethanol, further alterations in brain development exist. These include altered morphology of the midbrain-hindbrain boundary (MHB), reduced expression of *pax6a* in the eye (Zhang et al., 2017), forebrain, and hindbrain, as well as reduced expression of *epha4a* in rhombomere 1 of the hindbrain, which point towards altered brain development, including cerebellar development resulting from ethanol exposure. Further, early ethanol exposure has been shown to contribute to altered neurogenesis during early zebrafish development. Specifically, exposure corresponded with a clear reduction in differentiated neurons in the hindbrain and spinal cord (Joya et al., 2014). The defect was found to relate to populations of sensory neurons, where decreased cell proliferation and increased apoptosis was observed. Whilst these observations are interesting, it is not clear to what extent such alterations might contribute towards behavioural phenotypes and further investigation of the effects of developmental ethanol exposure on brain development are warranted.

## Postnatal Stress

### Postnatal Stress and Its Effects on Brain and Behaviour in Rodents

Within the rodent ELS field, the most widely used paradigms involve manipulation of the interactions of a female and her offspring during their early development. During early postnatal life, offspring homeostasis is dependent on care from the dam (Pryce et al., 2005). In infancy, postnatal days 3–14 are characterised as the SHRP in rodents. Isolation of the offspring from the dam for a sufficient period during this time is capable of stimulating the HPA axis in offspring, forcing a departure from the stress hyporesponsive state (Pryce et al., 2005). The most common rodent ELS protocols, known as maternal separation (MS) and maternal deprivation (MD), involve separating the pups from the dam one or more times during the SHRP (Sánchez et al., 2001). Rodent mothers exhibit naturally occurring variation in the quality of maternal care given to their pups in terms of licking/grooming and arched back nursing (LG-ABN) (Francis et al., 1999). As such, another ELS paradigm involves exposing the pups to either high or low quality maternal care via rearing with so-called high LG-ABN or low LG-ABN females. A different paradigm, the limited bedding/nesting (LBN) paradigm leaves dams with improper access to materials to build a satisfactory nest, which leads to maternal stress (Rice et al., 2008; Molet et al., 2014). As such the pups receive fragmented and erratic maternal care from the dam.

Many studies have demonstrated that impaired maternal care, in the form of MS, MD, LBN or low LG-ABN is associated with depressive-like symptoms in offspring such as increased immobility in the forced swim test (Pryce et al., 2005; Weaver et al., 2005; Franklin et al., 2010; Lajud et al., 2012; Amini-Khoei et al., 2017) and a reduced sucrose preference, indicative of anhedonia (Franklin et al., 2010; Bai et al., 2012; Gracia-Rubio et al., 2016; Amini-Khoei et al., 2017). Whilst some studies failed to demonstrate robust effects of ELS on behaviour in these paradigms (Pryce et al., 2005; Uchida et al., 2010), meta-analysis has suggested that MS is generally linked with increased depressive-like behaviours in rodents (Tractenberg et al., 2016). Rodent studies have also associated MS with increased anxiety-like behaviours in adult offspring (Pryce et al., 2005), and this has been supported with a meta-analysis (Tractenberg et al., 2016). However, the reported effects of ELS on anxiety-like behaviours are not entirely consistent, since a number of studies have reported no effects of ELS on anxiety-like behaviour (Lajud et al., 2012; Loi et al., 2014). In comparison with other behavioural endpoints, social behaviours have been much less well studied in rodents following ELS exposure. Some studies have suggested that prenatal, postnatal and juvenile stress are associated with disrupted social motivation in rodents (Pryce et al., 2005; Sandi and Haller, 2015) and unpredictable MS combined with unpredictable maternal stress (MSUS) has been shown to lead to impaired social memory, as measured by length of investigation of a familiar conspecific in male mice (Franklin et al., 2011). On the other hand, in rats, MS has been shown to have no effect on social recognition in a social recognition test (Hulshof et al., 2011). Meta-analysis also suggests MS in rodents is associated with memory performance impairments (Tractenberg et al., 2016). ELS in rodents has also been widely associated with impairment in cognitive functions such as learning and memory (Brunson et al., 2005; Hulshof et al., 2011; Suri et al., 2013).

In human studies, ELS and psychopathology have been associated with volumetric changes in specific regions of the adult brain associated with the stress response and cognitive function (Carballedo et al., 2012; Opel et al., 2014; Pechtel et al., 2014). However, similar structural changes in the hippocampus (Molet et al., 2016; Loi et al., 2017) and amygdala (Salm et al., 2004; Castillo-Gómez et al., 2017; Sarabdjitsingh et al., 2017) are less evident in rodent ELS models. ELS has also been associated with alterations in white matter structure in animal models (Sarabdjitsingh et al., 2017) and humans (McCarthy-Jones et al., 2018). In addition to these gross morphological changes, ELS is also associated with altered dendritic branching and spine density (Brunson et al., 2005; Koe et al., 2016), as well as reduced number of excitatory synapses (Wang et al., 2011), and alterations to hippocampal mossy fibers (Huot et al., 2002; Brunson et al., 2005) in rodent models. ELS can also lead to long-lasting changes in brain function (Sandi and Haller, 2015; Bick and Nelson, 2016; Teicher et al., 2016; Di Segni et al., 2018). One of the most widely studied effects of ELS on brain function is that of synaptic plasticity. In rodent models, ELS does not generally effect basal transmission, but alters long term potentiation in the hippocampus, in a manner that is region- and paradigm-specific (Derks et al., 2017). However, in male rats, ELS lead to amygdala hyperactivity in basal conditions and after a resident-intruder test in adulthood (Márquez et al., 2013).

In adulthood, the ELS-induced structural and functional changes in the brain are accompanied by molecular alterations, which may aid the maintenance of these changes. The most widely studied molecules that are implicated in the ELS-induced changes are cortisol, neurotrophins, neurotransmitters, neuropeptides and cytokines. Cortisol has been widely implicated as a mediator of the effects of ELS on adult neurogenesis, but its exact role is unclear (Kino, 2015; Lucassen et al., 2015; Odaka et al., 2017). Whilst ELS might transiently increase circulating glucocorticoid levels, and frequently lead to long-term alteration of basal or stress-induced HPA axis activity, the exact role of cortisol in mediating the effects of ELS on the brain requires further investigation. Indeed both increased and reduced cortisol levels have been associated with altered social behaviour in rodents (Sandi and Haller, 2015).

Further, the neurotrophic factor Brain Derived Neurotrophic Factor (BDNF) is implicated in mood and anxiety disorders and is upregulated by antidepressants (Fumagalli et al., 2007). ELS or acute adult stress have typically been associated with decreased expression of BDNF in the adult hippocampus (Nair et al., 2007; Suri et al., 2013), which often coincides with reduced neurogenesis. Indeed one of the clearest mechanisms by which ELS alters brain development is via altered neurogenesis, particularly in the hippocampus. Evidence suggests that various aspects of neurogenesis, neuronal development and brain patterning may be altered by ELS, including progenitor cell proliferation, neuronal differentiation and cell fate specification, cell survival, cell death (Korosi et al., 2012; Lajud and Torner, 2015; Bick and Nelson, 2016). These alterations may also contribute to changes in brain morphology, such as reduced hippocampal volume, as is sometimes observed in ELS-exposed individuals (Carballedo et al., 2012; Opel et al., 2014). ELS-induced changes to brain structure are also frequently accompanied by changes in brain function, such as to synaptic plasticity and neurotransmission (Bick and Nelson, 2016; Teicher et al., 2016; Derks et al., 2017). Neurotransmitter systems play a critical role in neurodevelopmental processes, neuronal plasticity and cognitive function. Indeed, the glutamatergic (Cameron et al., 1997), GABAergic (Earnheart et al., 2007; Martisova et al., 2012), dopaminergic (Flagstad et al., 2004), and serotonergic (Di Segni et al., 2018) systems are implicated in glucocorticoid- and stress-mediated effects on the brain and behaviour.

Diverse biological alterations following ELS are frequently connected to epigenetic changes that can regulate the expression of genes related to the HPA-axis, neuropeptides, synaptic plasticity and hormonal activity via the modification of DNA methylation, histone modification, and alteration in non-coding RNAs (McGowan et al., 2009; Roth et al., 2009; Bai et al., 2012; Kang et al., 2013; Ouellet-Morin et al., 2013; Zhang et al., 2015; Bahi, 2016; Bustamante et al., 2016; Tyrka et al., 2016; Williams et al., 2016; Wang et al., 2017). Accumulating evidence supports that GC signalling can induce a number of epigenetic modifications as well as regulating gene transcription (Zannas and Chrousos, 2017; Eachus and Cunliffe, 2018; Krontira et al., 2020). Early life exposure to cortisol can lead to a global decrease in methylation of DNA in some cases or local demethylation of cytosine-guanine dinucleotides (CpGs) in proximity to glucocorticoid response elements (GREs) in GC responsive genes (Bose et al., 2010; Wiench et al., 2011; Klengel et al., 2013; Resmini et al., 2016). The mechanisms underlying these alterations are not entirely clear. Some studies report that GC exposure promotes active- demethylation of CpGs and represses the maintenance/*de novo* creation of DNA methylation in a tissue-specific manner in a specific neural or developmental context, by regulating associated genes including Tet Methylcytosine Dioxygenases (TETs) and DNA methyltransferases (DNMT1, DNMT3a) (Benoit et al., 2015; Bose et al., 2015; Dong et al., 2015; Gassen et al., 2015; Sawamura et al., 2016). Histone modification patterns at GR binding sites can be modified via direct or indirect recruitment of histone modifiers such as acetyltransferase or deacetylase by GR (Di Stefano et al., 2015; Zannas and Chrousos, 2015; Vockley et al., 2016). Ultimately, those biological and epigenetic changes may contribute to an accumulation of risk for stress-related diseases over the lifetime via mechanisms affecting brain development and neural network formation during the early life stage (Zannas and Chrousos, 2017).

Recently, in a human hippocampal progenitor cell line, Provençal et al. (2020) identified substantial changes in long-lasting differentially methylated CpG sites (DMSs) following glucocorticoid exposure during neurogenesis. Interestingly, only a small number of lasting DMSs correlated with lasting changes in gene expression. However, 18.2% of transcripts that were mapped to long-lasting DMSs showed significant changes in gene expression after the additional acute challenge of dexamethasone treatment. These results indicate that a subset of the long-lasting DMSs may alter certain gene expression patterns throughout a lifetime by priming the GR response of neighbouring loci, rendering them more responsive to subsequent glucocorticoid exposure. Moreover, the researchers performed an enrichment analysis that detected a significant group of long-lasting DMSs that were conserved in peripheral tissues and could be used as a biomarker to predict maternal anxiety and depression in newborn cord blood samples.

Despite the significant connection between ELS and epigenetic modifications, it remains challenging to establish causal links between specific epigenetic modifications and biological alterations. Epigenetic modifications following ELS are varied depending on species, tissue or cell-type, type/intensity of stressor, exposure time-point, and duration of stress (Heim and Binder, 2012; Bock et al., 2015; Chattarji et al., 2015; Palma-Gudiel et al., 2015). Therefore, the broad impact of ELS on epigenetic mechanisms, diversity of developmental outcomes, and the heterogeneity of findings must be considered to establish definite epigenetic pathways following ELS. In addition, considerations such as cell-type specificity, individual variation, reverse causality or weak statistical power can make it difficult to draw significant conclusions regarding the role of epigenetic mechanisms in specific biological functions (Zilberman et al., 2007; Gertz et al., 2011; Lappalainen and Greally, 2017; Marzi et al., 2018) and findings are sometimes misinterpreted or over-generalised (Deichmann, 2020).

### Effects of Early Life Stress Exposure in Zebrafish

Early life stress may result from change in environmental conditions experienced by the animal in early life. Various stress protocols have been used in larval zebrafish, including changes in temperature, PH, water flow, restraint, netting, crowding, isolation, and predator exposure.

#### Water Movement

One seemingly important factor in modulating brain development and behaviour in zebrafish is movement during early life. During early development, restraint stress is associated with reduced progenitor cell proliferation in the zebrafish forebrain (Hall and Tropepe, 2018). In restrained animals, neuronal differentiation is increased at the expense of self-renewal, thereby depleting the progenitor pool. This, and to a lesser extent increased apoptosis, lead to smaller forebrain size in exposed larvae. In contrast, forced swimming during early life is associated with increased progenitor cell proliferation in the larval zebrafish pallium, and reduced neuronal differentiation (Hall and Tropepe, 2018). This finding suggests the opposite observation to that of restrained larvae: maintenance of an expanded progenitor pool at the expense of neuronal differentiation. In another study, an involuntary swim paradigm resulted in dysregulation of the HPA axis (Castillo-Ramírez et al., 2019). Here, in a later larval stage, animals that had experienced involuntary swimming during early development reconfigure their cortisol response to homotypic and heterotypic stressors. These results show how early life stress exposure can be linked to altered glucocorticoid function later in life in zebrafish.

Hall and Tropepe (2018) determined that it is physical cues associated with movement in early life that alter neurogenesis in the forebrain and these cues are conveyed by dorsal root ganglia. The mechanism by which movement affects neurogenesis in the zebrafish forebrain is not clear, although changes in cell cycling, cell fate and apoptosis may all play a part. Although glucocorticoids are known to modulate neurogenesis, it is not clear whether cortisol plays a direct role in movement-mediated alteration of forebrain neurogenesis or behaviour. As both restraint and forced swimming are expected to increase cortisol level, their differing effects on neurogenesis do not support a direct cortisol effect on neurogenesis in these paradigms. In another study, cortisol administration has been associated with increased progenitor cell proliferation in the early zebrafish forebrain and with behavioural alterations (Best et al., 2017). Increased forebrain proliferation is also observed after forced swimming (Hall and Tropepe, 2018), suggesting a potential role for cortisol in mediating movement-mediated alteration of forebrain neurogenesis. The difference in neurogenesis phenotypes following early life restraint or forced swim could reflect the different ways in which these paradigms modulate the HPA axis.

#### Social Isolation

The effects of social isolation during development on the brain and behaviour can be readily studied in zebrafish, since the lack of maternal care means that, unlike in mammals, the effects of social deprivation can be separated from other variables connected with maternal care, such as nutrition. The locomotor startle response is influenced by rearing environment in zebrafish: larvae raised in isolation exhibit a blunted startle response to a dark pulse in comparison to group-reared larvae, and this is thought to be attributable to a lack of tactile stimulation (Steenbergen et al., 2011). It has also been observed that socially isolated larvae exhibit enhanced social avoidance of conspecifics during social interactions, an effect mediated by lateral line inputs (Groneberg et al., 2020). Similarly, another study found that developmental isolation reduced social preference in juvenile fish and these fish were more anxious (Tunbak et al., 2020). In this study, isolation also lead to increased visual sensitivity and increased activity in the posterior tuberal nucleus of the brain, a region associated with the stress response, suggesting that in isolated fish heightened visual sensitivity leads to increased stress during social viewing, which may lead to the observed social aversion. On the other hand, another study found that unlike fish that were chronically isolated, developmentally isolated fish did not exhibit any changes in anxiety or social behaviours in adulthood (Shams et al., 2018). These data suggest that timing and duration of isolation may affect the behavioural outcomes.

A separate study also suggested that long-term isolation may affect the stress response, since isolation-reared zebrafish were found to have lower levels of dopamine and serotonin’s metabolite, 5-Hydroxyindoleacetic acid (5-HIAA), following exposure to an unpredictable chronic mild stress, when compared to group-reared fish (Fulcher et al., 2017), however, it was also observed that developmental isolation had no significant effects on baseline or stress-induced cortisol levels (Shams et al., 2020). Interestingly, in another study, developmentally isolated fish (isolated 0–7 dpf) were observed to have significantly reduced levels of DOPAC, but not dopamine, serotonin or 5-HIAA, when compared with social-reared fish in adulthood (Shams et al., 2018). Further, Varga et al. (2020) observed that social isolation of fish during a critical period of behavioural metamorphosis that occurs in the juvenile stage affects later behavioural responsiveness and serotonergic signalling in the dorsal pallium. Blockade of 5-hydroxytryptamine (5-HT) signalling was sufficient to prevent the serotonin-associated brain changes and behavioural deficits, suggesting that a serotonergic mechanism, sensitive to environmental manipulation during a specific time window, modulates context-dependent behaviours in later life.

Long-term isolation of zebrafish, such as from 6 dpf until 6 months, can have profound effects on brain development in a region-specific manner. Lindsey and Tropepe (2014) showed that isolated fish exhibited a significant reduction in proliferation in sensory niches of the brain, but not in the telencephalon and that these changes could not be consistently rescued by exposure to social novelty. An interesting recent study shows that social isolation reduces the expression of neuropeptide *pth2*, and remarkably a brief exposure of isolated fish to a social group can rescue this (Anneser et al., 2020).

#### Chemical Exposure

One route by which activation of the HPA axis may occur is by exposure to chemicals. A large number of studies have tested the effects of exposure to chemical substances in early development on the brain and behaviour in zebrafish. These toxicological studies have relevance both as models of the adverse effects of chemical exposure in humans and as studies of the effects of such chemicals on fish, since human actions can cause certain chemicals to contaminate aquatic environments. This vast amount of literature is outside of the scope of this review and the readers are referred elsewhere (Hill et al., 2005; Dai et al., 2014; Lee et al., 2015). Instead, here we will mention two exemplary studies testing the effects of chemicals that alter the HPI axis.

Bisphenol A (BPA) is commonly used in the production of plastics and other consumer products and in rodent models prenatal exposure to BPA is associated with the development of behavioural challenges in later life (Kinch et al., 2015). Treatment of embryonic zebrafish with BPA results in precocious neurogenesis in the developing hypothalamus, as well as hyperactivity during the larval stage (Kinch et al., 2015). This work identified an unexplored pathway through which BPA exerts its effects on neurogenesis and behaviour: via androgen-receptor mediated up-regulation of aromatase (Kinch et al., 2015). It is not clear, however, how altered aromatase activity might affect neurogenesis and behaviour. In other animals, BPA exposure is known to cause dysregulation of the HPA axis (Panagiotidou et al., 2014). Given that cortisol is known to affect neurogenesis and behaviour in the larval zebrafish (Best et al., 2017), and that cortisol is known to interact with sex steroids, this may represent a downstream effect of BPA exposure.

Another example that has been studied in zebrafish is exposure to fluoxetine, a selective serotonin reuptake inhibitor (SSRI), which is widely prescribed to treat depression in humans. Fluoxetine alleviates symptoms of depression by inhibiting 5-HT reuptake transporters on presynaptic neurons, thereby enhancing serotonergic neurotransmission (Stahl, 1998). However, concerns have been raised regarding the effects of exposure to fluoxetine during developmentally sensitive periods (Suri et al., 2015), such as exposure to the foetus when taken by pregnant women, and exposure to children and adolescents, to whom fluoxetine may also be prescribed (Jane Garland et al., 2016). Fluoxetine is widely prescribed globally and following excretion can enter aquatic environments where variable concentrations have been quantified in different water bodies (Parolini et al., 2019).

Exposure to fluoxetine during early life in zebrafish is associated with modulation of stress-associated genes and key neurotransmitter transporters in larvae (Parolini et al., 2019). These changes correlate with a reduced locomotor response to a touch stimulus. Early life exposure to fluoxetine also has long-term effects on the HPA axis and behaviour in zebrafish and these effects depend on the dose and timing of exposure, and are also sex-specific. Exposure during embryonic – early larval, larval, or juvenile stages lead to a blunted stress response in adult males. Additionally, the reduced cortisol levels resulted in reduced locomotion and exploratory behaviour following embryonic-early larval stage and larval fluoxetine exposures, but not following juvenile exposure (Vera-Chang et al., 2018). Meanwhile fluoxetine exposure during juvenile stages lead to reduced basal cortisol in females but not males (Vera-Chang et al., 2019). Further, in adult females, exposure during the juvenile, but not larval stage resulted in increased locomotion and exploratory behaviour.

The observed hyporeactivity of the stress response following early life fluoxetine exposure in zebrafish is consistent with observations in children prenatally exposed to SSRIs (Oberlander et al., 2008) and chronic blunting of basal cortisol levels has been linked with a number of psychological disorders in humans (Wichmann et al., 2017). One of the most intriguing findings from the studies of fluoxetine exposure, is that the suppression of cortisol levels and altered behaviour persists for multiple generations in unexposed fish (Vera-Chang et al., 2018). The HPA axis and behavioural defects may well be mediated by epigenetic alterations that in some cases are stably transmitted across generations, as has been previously observed following stress (Babenko et al., 2015). Indeed, 5-HT is known to alter GR expression via epigenetic modifications. The zebrafish data suggests that cortisol level maybe responsible for the observed behavioural alterations (Vera-Chang et al., 2018). Whether fluoxetine exposure alters behaviour in zebrafish via epigenetic modification of GR remains unknown. The analyses of the effects of fluoxetine exposure on the HPA axis and behaviour in zebrafish seem to be consistent with findings in rodent and human studies. Given the rapid external development of the early zebrafish and lack of maternal care, it is an advantageous system with which to model the effects of prenatal fluoxetine exposure in humans.

#### Other Stressors

Brief exposure to anoxia during embryogenesis has also been shown to have profound and long-term effects on zebrafish behaviour. Anoxia-exposed zebrafish develop to become more dominant, exhibit more aggressive behaviours and have increased testosterone levels (Ivy et al., 2017). Interestingly, exposure to mild stress during early life may confer seemingly advantageous behavioural changes. In one study, zebrafish that were exposed to a combination of environmental enrichment and daily net chasing exhibited enhanced learning and reduced anxiety when tested as juveniles, and the reduction in anxiety behaviour was apparent even when fish were tested in adulthood after returning to standard housing for several months (De Pasquale et al., 2016).

#### Combined Stressor Exposure

In addition to acute and chronic exposures to individual stressors, paradigms are also established that use exposure to a combination of different stressful stimuli to induce ELS, such as chronic unpredictable early life stress (CUELS) (Fontana et al., 2020). Larvae exposed to a 7 day CUELS protocol were observed to have reduced anxiety levels as adult fish, meanwhile a 14-day exposure had the opposite effect (Fontana et al., 2021). The CUELS paradigm did not affect memory and cognition or social behaviour. On the other hand, exposure of juvenile fish to combined stress for 3 days had a positive effect on working memory in adulthood, while having no effect on shoal cohesion or anxiety behaviour. Interestingly, zebrafish exposed to a chronic unpredictable stress paradigm during the larval stage exhibited anxiety-like behaviours in the days following stressor exposure, but these effects were transient (Golla et al., 2020). These studies indicate that the timing and duration of exposure, as well as timing of sampling will influence the effects of ELS on behavioural outcomes.

### Effects of HPA Axis Manipulation

Whilst environmental manipulations have been used to study the effects of ELS on the brain and behaviour, it is hard to identify the precise contributions of individual HPA axis components to the phenotypic outcomes in these studies. This question can be addressed by manipulation of the individual molecular players via genetic, optogenetic or pharmacological manipulation. Most studies have focused on uncovering the effects of increased level of glucocorticoid, since cortisol can be readily quantified in the zebrafish.

#### Genetic Manipulations

The genetic tractability of the zebrafish has made it an attractive model organism for some time and the availability of CRISPR-Cas9 technology in recent years has extended this further (Albadri et al., 2017). Zebrafish lines with stable mutations that disrupt individual components of the HPA axis have been created and have begun to shed light on the development of the HPA axis and its role in brain development and behaviour, across the life-course.

One of the most widely studied HPA axis molecules is the GR, one of two receptors via which cortisol exerts its actions including the negative feedback on the HPA axis. A number of different mutants for *nr3c1*, the gene encoding the GR, have been created, and used to study a variety of processes, including development of the HPA axis and behaviour. Loss of GR function means that negative feedback to the HPA axis is compromised and thus leads to high basal cortisol levels in both larvae (Griffiths et al., 2012; Facchinello et al., 2017; Faught and Vijayan, 2018) and adult fish (Ziv et al., 2013) and the HPA axis is not responsive to an acute stressor (Ziv et al., 2013; Facchinello et al., 2017; Faught and Vijayan, 2018).

A number of studies have investigated the role of *nr3c1* in behaviour, using various assays and the results of the different studies have sometimes conflicted. Reduced general locomotion has been widely reported in both larvae (Griffiths et al., 2012; Sireeni et al., 2020) and adult (Ziv et al., 2013; Sireeni et al., 2020) GR mutant fish in some studies, but not all (Faught and Vijayan, 2018). The first reports on a GR mutant showed that in larval stages mutants displayed a heightened startle response, in that they would not habituate to repeated stimulus exposure (Griffiths et al., 2012), and that as adults, they displayed freezing behaviour and an avoidance of the walls of a novel environment (Ziv et al., 2013), which the authors likened to depressive-like behaviour.

However, subsequent analysis of the same mutant revealed that mutant larvae exhibited a similar avoidance of the walls and confirmed the freezing behaviour in adulthood, however, this was accompanied by increased thigmotaxis in adulthood (Sireeni et al., 2020), the opposite effect to that observed previously. In contrast to the study of Ziv et al. (2013), the authors suggested that these behaviours were indicative of increased anxiety in fish lacking GR activity. An analysis of anxiety-like behaviour in larvae, however, revealed no significant effects, suggesting that in GR mutants, anxiety behaviour may only become apparent in adulthood. Further to the effects of loss of GR function on general locomotion and anxiety-like behaviour, a lack of feeding entrainment has also been reported in juvenile and adult GR knockout zebrafish (Morbiato et al., 2019). Whilst the authors focused on the requirement for GR in circadian rhythm, they concluded that clock gene expression was not responsible for the altered behaviour. This leaves the interesting possibility that other alterations in brain development or function such as alterations of the motivation system might be responsible for this behavioural change.

These studies of loss of GR function have highlighted the role of GR in anxiety- and depressive-like behaviours across the life course. In some cases, the research has also shown that the increased cortisol level resulting from loss of GR function is not directly responsible for the observed behavioural abnormalities: while behavioural defects can be normalised via treatment with fluoxetine, this is not accompanied by a reduction in cortisol level (Griffiths et al., 2012; Ziv et al., 2013). This suggests that a defect in serotonergic function in the brain of GR mutants, perhaps downstream of cortisol, is responsible for the observed behavioural defects. Indeed, glucocorticoids are known to influence the serotonergic system and both are implicated in the aetiology of affective disorders (Lanfumey et al., 2008). Despite this initial insight into the mechanism underlying behavioural defects in the GR mutant zebrafish, a comprehensive analysis into the effect of loss of GR function on the brain, including the serotonergic system, is lacking.

In contrast to GR, much less is known about the effect of MR manipulation in zebrafish. However, a recent analysis of MR^–/–^ in zebrafish reveals that MR is involved in stress-related behaviour, as knockout animals fail to exhibit glucocorticoid-mediated larval hyperactivity to a light stimulus (Faught and Vijayan, 2018). Meanwhile in another study, MR^–/–^ larvae exhibited no difference in their rapid locomotor response to an acute stressor, although the authors questioned whether they had achieved a complete loss-of-function in their MR knockout line (Lee et al., 2019). Further work is needed to establish whether MR plays a role in modulation of additional behaviours in zebrafish, such as anxiety-like behaviour, as is the case in mice (Rozeboom et al., 2007).

#### Pharmacology

The HPA axis can also be manipulated via direct targeting of pharmacological agents. The most commonly used are synthetic cortisol and dexamethasone, both agonists of the GR. Stimulation of GR in early life initiates negative feedback to the HPA axis, leading to a reduction in basal cortisol levels (Wilson et al., 2013, 2016). In contrast, fish that were exposed to GR agonists in early life that were subsequently raised under normal conditions, exhibit normal basal cortisol levels but show a heightened HPI axis response to an acute stressor (Wilson et al., 2016).

Dysregulation of the HPI axis using GR agonist treatment in early life is also associated with behaviour changes in zebrafish. Whilst dexamethasone exposure in early life has not been associated with any effects on basal locomotion in zebrafish larvae (Steenbergen et al., 2011; Wilson et al., 2013, 2016), cortisol treatment has often been shown to cause hyperactivity (Best and Vijayan, 2017; Faught and Vijayan, 2018). Additional behavioural phenotypes in larvae include altered thigmotaxis in an open field test (Wilson et al., 2013; Faught and Vijayan, 2018), and a reduced locomotor response to an acute stressor (Steenbergen et al., 2011). Whilst few studies have investigated the long-term consequences of early life exposure to GR agonists such as dexamethasone in zebrafish, the results of initial studies are confusing. Whilst some reported increased anxiety-like behaviour, as measured in the novel tank diving test (Khor et al., 2013; Kumari et al., 2019), others, using the same assay, reported the opposite (Wilson et al., 2016). This conflict may be caused by the different dosing regime, which would suggest that varying the timing and duration of exposure could have profoundly different effects on behaviour. However, this notion requires further testing.

These studies highlight the utility of the zebrafish to uncover behavioural alterations following dysregulation of the HPI axis in early life. Initial studies suggest that cortisol signalling via MR, rather than GR plays an important role in regulating cortisol-induced hyperactivity following early life exposure (Best and Vijayan, 2017; Faught and Vijayan, 2018). Beyond this, the mechanisms underlying behavioural alterations following HPA axis dysregulation in early life remain elusive and the effects of such treatments on the brain have not yet been investigated.

Several studies also examined the effect of GR antagonist, mifepristone treatment in zebrafish. Early life exposure to mifepristone was observed to reduce basal locomotor activity in exposed larvae (Wilson et al., 2013). An additional study observed that, as in an *nr3c1* mutant, mifepristone significantly decreased the locomotor response to an abrupt challenge in larvae (Lee et al., 2019). Further studies are required to establish whether early life mifepristone treatment has long-term effects on the brain and behaviour in zebrafish.

#### Optogenetics

Optogenetics has great potential within the zebrafish field, due to the transparent nature of the larval body, rendering its tissues accessible to light. Tissues that are too small to be amenable to surgical manipulation can be non-invasively manipulated using cell type specific promoters, and this manipulation is aided by the genetic tractability of the zebrafish. Using optogenetic techniques to selectively modify the level of individual HPI axis components with a high degree of temporal precision has recently been demonstrated using the zebrafish larva (De Marco et al., 2016, 2013; Gutierrez-Triana et al., 2015).

**FIGURE 1:**
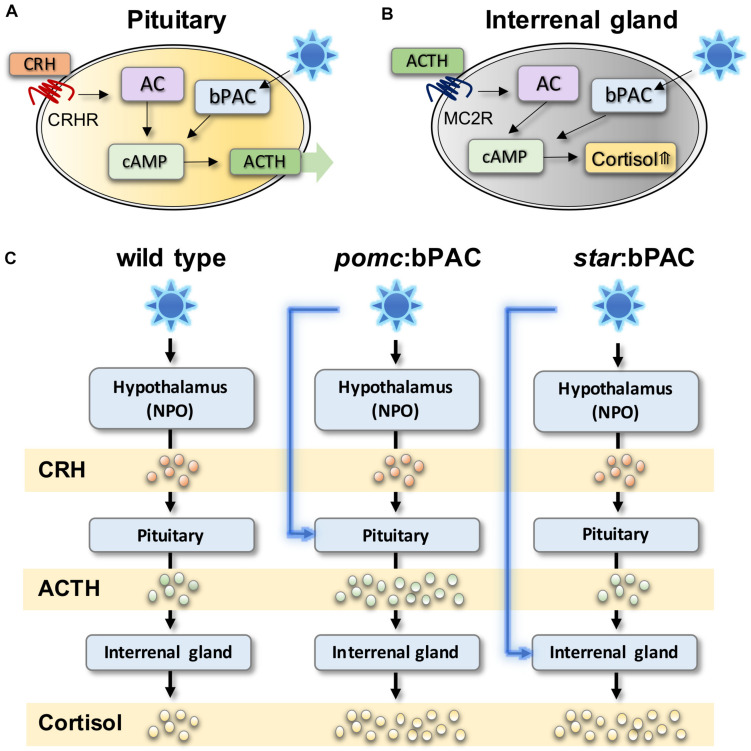
Optogenetic modulation of stress hormones in zebrafish can be used to study acute and long-term effects of HPI axis modulation. **(A)** In pituitary corticotroph cells, bPAC activation via blue light exposure activates cAMP signalling, which is expected to amplify ACTH release from the pituitary. **(B)** In steroidogenic cells bPAC activation by blue light activates cAMP signalling which leads to increased level of cortisol. **(C)** In transgenic *pomc*:*bPAC* larvae, exposure to blue light leads to hyperactivation of the HPI axis at the level of the pituitary, and also the interrenal gland, since increased bPAC-induced ACTH release drives increased cortisol release, meanwhile targeting bPAC to steroidogenic cells of the interrenal gland in *star*:*bPAC* larvae leads to overproduction of cortisol only, under blue light exposure. Adapted from Gutierrez-Triana et al. (2015), De Marco et al. (2016).

De Marco et al. (2013) first demonstrated the technique in zebrafish by manipulating pituitary signalling. Using a cell-type specific promotor, *beggiatoa* photo-activated adenylate cyclase (bPAC) was targeted to corticotroph cells of the anterior pituitary that produce ACTH (Figure 1A; De Marco et al., 2013). Exposure of these transgenic larvae to blue light, or white light with a blue component, leads to increased cAMP levels specifically within bPAC-positive pituitary corticotroph cells. In pituitary corticotroph cells, cAMP increase causes release of ACTH, and thus in bPAC-positive larvae light exposure leads to ACTH-induced secretion of cortisol (Figure 1C). Using a similar technique, transgenic zebrafish lines have also been created in which bPAC is targeted to Steroidogenic acute regulatory protein (*StAR)*-expressing interrenal cells (Gutierrez-Triana et al., 2015; Figure 1B). In these larvae, blue light causes an increase in cAMP in steroidogenic interrenal cells, which stimulates cortisol synthesis (Figure 1C).

These studies demonstrated that such transgenic zebrafish lines can be used to study rapid non-genomic effects of hormone signalling on behaviour in free-swimming larvae (De Marco et al., 2016). Additionally, these transgenic lines can be used to study the delayed and long-term effects of early life stress, since exposure to blue light could induce hypercortisolaemia in transgenic larvae. The ability to specifically and precisely manipulate the level, duration and timing of HPA axis activation will allow critical questions in the ELS field to be addressed, such as how the exact GC level, exposure duration and developmental timing of exposure affects the brain and behaviour. This technique could also be combined with imaging and with high-throughput behaviour assays in zebrafish larvae to identify the effects of ELS on brain structure and function. However, it should be noted that the use of these cell-type specific promotors does not necessarily mean specific manipulation of individual HPA axis hormones. Indeed other molecules that co-localise to the same cell types manipulated in these studies may also be altered via light exposure and contribute to the phenotypic outcome.

## Outlook and Concluding Remarks

Significant progress has been made in recent decades in our understanding of how ELS affects brain development and behaviour largely thanks to rodent models. Yet establishing robust mechanistic links between early life stress exposure and resultant phenotypes has been a challenging task. For example, in prenatal stress paradigms, it is difficult to assess the level of direct stress exposure experienced by foetus when mother is stressed. In postnatal ELS paradigms such as the MS protocol, inconsistent effects are often observed due to various factors including strain differences in maternal care, inter- and intra-litter variations in maternal care, and the multifaceted nature of the MS treatment, etc. (Pryce et al., 2005).

As an ELS model, the zebrafish has begun to gain traction over the past decade. As a model for studying human disease, the zebrafish represents a good balance between relative simplicity and homology with humans. Whilst the status of the current zebrafish ELS field is characterised by breadth rather than depth, the zebrafish model has the potential to complement and extend mammalian studies, to tackle some of the long standing open questions in the ELS field including:

### Comprehensive Brain-Wide Mapping of ELS-Induced Molecular and Functional Alterations

The effects of ELS on brain development using rodent models have largely been studied in the context of altered neurogenesis in the hippocampus. ELS-induced behavioural alterations include cognitive and affective domains, and it is likely that ELS leads to brain-wide alterations in many stress-sensitive regions. The small-sized and transparent zebrafish brain combined with a battery of imaging and genetic tools ideally suit such network-level analysis.

### Comprehensive Mapping of the Developmental Trajectory of ELS-Induced Changes Into Adulthood

External fertilisation and rapid early development allow mapping of the relationship between stimulus exposure and resultant changes throughout development. Larvae can be raised in a tightly controlled environment where the consequence of alteration of a given variable could be tracked through multiple stages in development.

### Linking Gene Functions and Behaviour

External development with ease of genetic access means zebrafish can be used to easily study gene-environment (GxE) interactions, which is a key feature underlying many psychiatric diseases (Kendler and Eaves, 1986; Cadoret et al., 1995; Heim and Binder, 2012). ELS-linked psychiatric diseases such as depression are typically polygenic in nature and studying the loss-of-function phenotypes of many genes together or in parallel is much cheaper and easier in zebrafish than in a mammalian system. Indeed, a recent study characterising 132 human schizophrenia-associated genes exemplify a large scale genotype-phenotype study possible in zebrafish (Thyme et al., 2019).

### Drug-Discovery

The small size of the zebrafish larva makes it an ideal organism to use in high throughput drug screening using an intact animal (Wiley et al., 2017). A common feature of ELS-induced psychiatric disorders and preclinical ELS models is long-term alteration of the stress regulating system (Chrousos, 2009; Danese and McEwen, 2012; Heim and Binder, 2012; Doom and Gunnar, 2015). Despite this, no current treatment for psychiatric disorders specifically targets the HPA axis. Here, the zebrafish system has the potential to contribute due to its utility as a powerful high-throughput drug-screening tool.

Cellular and molecular mechanisms induced by ELS that alter brain development and drive the structural and functional changes in the adult ELS-exposed brain remain poorly understood. One of the ultimate questions in behavioural neuroscience is how molecular changes facilitate behavioural alterations. In the ELS field, few studies have achieved such a feat so far, i.e., linking early life molecular mechanisms with altered behaviour in adulthood. Making convincing connections between the two is not at all trivial, yet the zebrafish model offers many important advantages to tackling this important challenge.

## Author Contributions

HE: writing of the original draft. M-KC, HE, and SR: writing, review, and editing. SR: funding. All authors contributed to the article and approved the submitted version.

## Conflict of Interest

SR holds a patent, European patent number 2928288 and US patent number 10,080,355: “A novel inducible model of stress.” The remaining authors declare that the research was conducted in the absence of any commercial or financial relationships that could be construed as a potential conflict of interest.
